# Long-Term Potentiation of Prelimbic Cortex Ascribed to Heat-Sensitization Responses of Moxibustion

**DOI:** 10.1155/2019/9465181

**Published:** 2019-07-25

**Authors:** Rixin Chen, Zhimai Lyu, Dingyi Xie, Dandan Huang, Yanjun Chen, Chunmei Wu

**Affiliations:** ^1^Affiliated Hospital of Jiangxi University of TCM, Nanchang 330006, Jiangxi Province, China; ^2^First Affiliated Hospital of Gannan Medical University, Ganzhou 341000, Jiangxi Province, China; ^3^Gannan Medical University, Ganzhou 341000, Jiangxi Province, China; ^4^Jiangxi University of TCM, Nanchang 330006, Jiangxi Province, China

## Abstract

Heat-sensitization responses occurred in certain patients while exposed to suspended moxibustion. The response often indicated that the efficacy of moxibustion to those with it tended to triumph over those without. However, its mechanism remains to be explained. Our previous fMRI and EEG studies confirmed the changes of activities in cerebral certain regions accompanied with heat-sensitization responses, especially in prefrontal cortex. Therefore, we hypothesize that neurological system is involved in moxibustion-induced heat-sensitization responses. In the present study, phosphorylation of Cofilin representing long-term potentiation in synapse of prelimbic cortex of medial prefrontal cortex in stroke rats over suspended moxibustion was assessed, and the size of phosphorylated Cofilin positive spine in synapse was also measured. The result showed that heat-sensitization responses were observed to augment cerebral ischemic stroke-induced phosphorylation of Cofilin in prelimbic cortex of rats and increase the numbers of large synapses. This indicated that long-term potentiation of prelimbic cortex was attributed to heat-sensitization responses that were certain neurological responses of medial prefrontal cortex to suspended moxibustion.

## 1. Introduction

Moxibustion is a traditional Chinese medicine approach to healing, with burning dried plant materials (*Artemisia moxa*), achieving the alleviation of pain suffering and the cure of disease. It can be applied with heat generated from burning* moxa *3–5 cm above over skin surface (suspended moxibustion, Supplementary Figure S1). One of suspended moxibustion exercises is termed heat-sensitization moxibustion that we have reported previously [1]. During the process of heat-sensitization moxibustion, the recipient's reaction or self-perception is termed heat-sensitization response or phenomenon in which the recipient significantly relieved the suffering in many cases, being comforted by sensing enlargement of intensity and space of heat that was not from moxa burning radiation (rather than the heat moxibustion-radiated). Heat-sensitization responses are considered the stimulation and operation of meridian QI in human body, which demonstrates great efficiency of moxibustion [2]. However, there is little known about the mechanism. Conventional understanding of nerve conduction pathways has not recounted the heat-sensitization responses of moxibustion, especially heat transfer or heat expansion induced by no-radiation heat. In our previous studies, most reactions to heat-sensitization moxibustion, which were described by the participants, were observed directly or collaterally relating with neurological system; noticeably the brain perception was spotted [3]. A study of functional magnetic resonance imaging (fMRI) also showed that heat-sensitization response was manifested in the changes of brain activities in prefrontal cortex [4]. Therefore, we hypothesize that neurological system is involved in moxibustion-induced heat-sensitization responses.

Through our clinical observations, moxibustion-induced heat-sensitization responses vary with the state of a disease [1]. We consider the variation associating with neuroplasticity that also has variable characteristics, so that study into synaptic neuroplasticity may reveal the mechanism. Long-term potentiation (LTP) is a sign of synaptic neuroplasticity, which was* in vivo* marked by the increase of phosphorylated Cofilin (pCofilin) in the spines of synapse [5, 6]. On that account, phosphorylation of Cofilin in synapse indicates neuroplasticity in a target area. Based on the above theories and our previous study that showed the tail midpoint temperature rose when cerebral ischemic stroke rats were exposed to suspended moxibustion exercise [7], in this study we investigated synaptic neuroplasticity in prelimbic cortex (PrL) of medial prefrontal cortex (mPFC) of rats over suspended moxibustion exercise, illuminating the relationship between heat-sensitization response and neuroplasticity of mPFC.

## 2. Materials and Methods

### 2.1. Animals and Experimental Design

50 mature male Sprague-Dawley rats (220 to 250 g) were purchased from Shanghai Slaccas Laboratory Animals Co., Ltd., in Shanghai, China. They were housed in the cage at temperature 20~25°C and humidity 40~70%. The rats were habituated to the cage for 6 days prior to test. Then they were randomly stratified into 4 groups.

1. Normal Group (N, n=10)
2. Sham-Operated Group (S, n=10)
3. Ischemic Control Group (C, n=10)
4. Ischemia over moxibustion exercise Group (M, n=20)

The usages of animal in the experiments were conducted in accordance with NIH Guidelines and approved by the Animal Use and Care Committee for Jiangxi University of TCM for Scientific Purposes.

### 2.2. Experimental Stroke in Rats

The rats were injected intraperitoneally with Sodium Pentobarbital (3%) at a dose of 30 mg/kg to be anesthetized. Core body temperature was monitored using a rectal probe and maintained at 37±0.5°C by heating lamp and a heating pad. The values of pH, PaO_2_, PaCO_2_ of arterial blood gas, and blood pressure were closely monitored via catheterizing the right femoral artery. The Middle Cerebral Artery Occlusion (MCAO) was obtained by the Intraluminal Filament method as previously described [8]. A fishing line (Simago Fishing Tackle Company) of 0.205 mm in diameter and 5 cm in length with a rounded tip was inserted through the common carotid artery and gently advanced to the origin of the middle cerebral artery. The rats underwent occlusion for 2 hours, and then the fishing line was withdrawn to allow for reperfusion. Sham-operated rats were treated in the same way, but the MCA was not occluded. Adequacy of vascular occlusion and reperfusion were assessed by Laser Doppler Monitoring of cerebral cortical perfusion. The exclusion criteria were as follows: (a) death within 24 h after tMCAO, (b) neurological severity score Longa=0, and (c) subarachnoid hemorrhage as macroscopically assessed during brain biopsy. Subsequently, 1 out of the 10 ischemic control rats (1 death) and 2 out of the 20 ischemic rats with moxibustion exercise (1 death and 1 score=0) were excluded. In total, 47 out of 50 rats were used for analysis.

### 2.3. Temperature Measurement in the Tail of Rats over Moxibustion Exercise

Rats were treated with suspended moxibustion exercise in this study, precisely, hanging moxa strip at 3 cm higher than the target area, untouched upon the recipient. Moxa strip made of moxa powder was 12 cm in length, 0.6 cm in diameter, provided by the Affiliated Hospital of Jiangxi University of TCM, China. The fur on the rats neck was shaved off to uncover the acupoint dà zhuī (DU14) that is the location of the seventh cervical vertebrae; DU14 is considered very important for brain functions in acupuncture and moxibustion philosophy [9]. The rats were separately placed in a purpose-built cage and situated in a comfortable position, easing the rat's tension and facilitating suspended moxibustion exercise. Room temperature was consistent at 25±2°C through the entire experimental process. DU14 was exposed to suspended moxibustion exercise for 30 minutes; that treatment was once a day for 3 days (Supplementary Figure S2). The initial suspended moxibustion exercise was exerted to the rats six hours afterwards reperfusion. The temperature was taken on the rat's midpoint tail, precisely by an electrodigital thermometer (Shanghai Medical Instrument Factory, Shanghai, China). The initial record for temperature was when the rats were placed quietly in the designed cage for 10 minutes, followed by 5-minute interval measurements for 7 times. Based on the change of rats tail temperature, the group M was divided into two subgroups, a non-tail temperature increasing subgroup (≤1°C an average of 3 days, non-TTI) and a tail temperature increasing subgroup (>1°C an average of 3 days, TTI).

### 2.4. Immunocytochemistry Examination

Rats were given halothane to be anesthetized and then perfused with 4% paraformaldehyde in 0.1 M sodium phosphate buffer (PB), pH 7.2. Brains were postfixed for 2 h, cryoprotected in 20% sucrose/PB, and then sectioned (25*μ*m) on a freezing microtome. The position of the slices was based on Bregma 2.76mm. Free-floating sections were processed for double-immuno-labeling that rabbit anti-Cofilin (1:100, ab-42824, Abcam) or anti-pCofilin [pS3] IgG (1:100, ab-12866, Abcam), and mouse anti-PSD-95 (1:200, MA1-045, Thermo Fisher Scientific) were used as the first antisera, and Alexa Fluor 555 anti-rabbit IgG and Alexa Fluor 488 anti-mouse IgG (both 1:200; Invitrogen, Carlsbad, CA) in PB as the secondary antisera. Control tissue was processed by the same procedures but without the first antisera. Immunocytochemistry for pCofilin and PSD-95 was examined to three batches of tissue, and each batch was simultaneously processed, and each group contained 2-4 samples. All the groups N, S, C, non-TTI, and TTI contributed their samples to test. Every batch contained tissue from the group C, and quantification of immunoreactive puncta within a batch was normalized to the mean value of the group C for that batch.

### 2.5. Confocal Microscopy and Three-Dimensional Reconstruction

Laser scanning confocal microscopy was applied to take an image of PrL of mPFC in unaffected hemisphere. LSM 880 Axio Observer (Carl Zeiss AG, Jena, Germany) and a Plan Apochromat 63x/1.40 Oil DIC M27 objective were employed. Optical section is 0.6*μ*m thickness and the image covering a 101.41 × 101.41 *μ*m area within PrL of mPFC in unaffected hemisphere of 38 sections for each rat whereby more than 300,000*μ*m^3^ (area×0.6*μ*m optical section thickness) was examined for each rat. Images were analyzed with Carl Zeiss ZEN 2.1 (blue edition) (Carl Zeiss AG, Jena, Germany). Tissue in each batch was parallel scanned. Analysis was conducted blind to sample identity on batches that had been processed together. Identified objects < 0.04 *μ*m^2^ and > 1.2*μ*m^2^ were excluded from analysis. Counts from multiple sections were averaged to produce a representative value for each rat. Three-dimensional reconstructions of the target field were generated and qualitatively analyzed with Carl Zeiss ZEN 2.1 (blue edition) for overlap between pCofilin and PSD-95-immunoreactive elements.

### 2.6. Statistical Analysis

**Figure 1:**
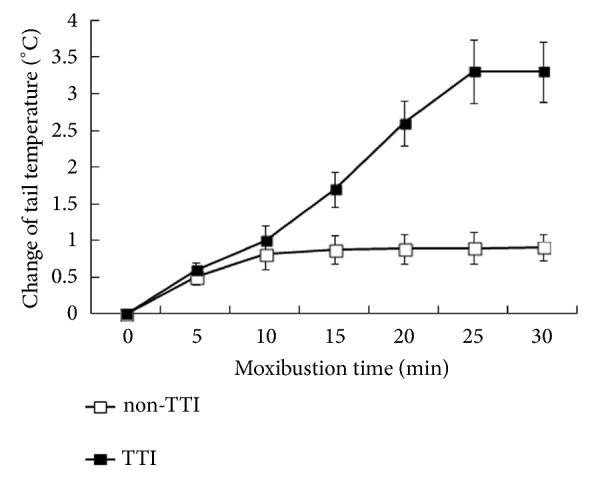
Change in tail temperature induced by suspended moxibustion exercise on the acupoint DU14 in MCAO rat model. Because the change of tail temperature was similar among the three consecutive testing days, data of the first day were presented as a representative. Data were expressed as mean ± SD.

Data was analyzed with a* t *test for two groups and one-way analysis of variance (ANOVA) with post hoc Newman–Keuls multiple range test for multiple groups. SPSS 20.0 was used for analysis. Significance was determined at* P*<0.05. The values in Figure 1 were reported as the mean± SD, and other values were reported as the mean ± SEM in the figures.

## 3. Results

### 3.1. Temperature Increase in the Tail of Rats with Cerebral Ischemic Stroke

All the rats receiving moxibustion exercise showed very quiet instead of irritated during the moxibustion treatment. Among the 18 rats of group M, 11 rats showed the tail temperature increased more than 1°C (3°C on average), and 7 rats were seen at the tail temperature increase ≤1°C on average. Therefore, there were 11 rats in TTI subgroup and 7 rats in non-TTI subgroups. The incidence of tail temperature increase was similar to that of the previous experimental results [10]; see Figure 1.

### 3.2. Cofilin-Positive Spines Contain a Small Fraction of pCofilin-Positive Spines in the Dendritic Spines

**Figure 2:**
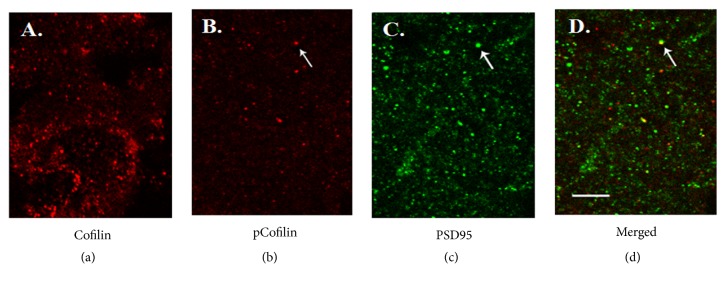
Cofilin, pCofilin, and PSD-95 immunostaining in PrL of mPFC. (a) Cofilin immunoreactivity was abundant in discrete puncta but was absent from cell bodies (data not shown) and dendrites. (b) PCofilin immunoreactivity puncta were present in smaller numbers compared to those labeled for total cofilin (a). (c) PSD-95 immunoreactivity profiles were numerous. (d) Merged images show that pCofilin immunoreactivity profiles were associated with PSD-95 immunoreactivity puncta. The arrows point to the same sites in (b)–(d) to show spatial relationship and overlap. Scale bar: 5 *μ*m.

The change of synaptic structure requires the participation of structural protein actin fiber, and the change of action fiber is regulated by Cofilin. The Cofilin protein that is mostly restricted to dendritic spines removes actin monomers from the sharp end of growing actin filaments. The phosphorylation of Cofilin suppresses this activity [6]. Immunocytochemistry examination showed that Cofilin immunoreactivity was abundant throughout all PrL slices; see Figure 2(a). Spines with dense pCofilin immunoreactivity were present in only a small fraction of densely Cofilin immunoreactivity spines; see Figure 2(b). Localization of pCofilin to spines was confirmed by its juxtaposition with structures that were densely immunoreactive for the integral postsynaptic density protein PSD-95; see Figures 2(b)–2(d).

### 3.3. Increase of pCofilin in Synapse of PrL Cooccurring with the Temperature Increase of Rat Tail

**Figure 3:**
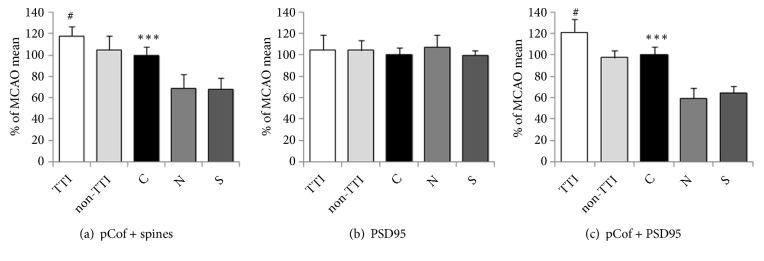
MCAO model increased the number of pCofilin positive (pCof+) spines, and moxibustion-induced heat-sensitization responses in rat's model characterized by tail temperature increase had enhanced this effect. PCof+ spines were counted and values for a rat in a given cohort were normalized to the mean score for ischemic control rats in that cohort. (a) MCAO rats either or not treated with suspended moxibustion exercise (TTI, non-TTI, and C groups) had more pCof+ spines than did normal rats (N group); this effect was absent in sham rats (S group); differences in pCof+ puncta between TTI versus non-TTI or C groups were significant. (b) The increase in numbers of pCof+ puncta in MCAO rats either or not treated with suspended moxibustion exercise was not accompanied by an increase in total number of PSD-95 puncta. (c) The number of PSD-95 puncta that were colocalized with pCofilin immunoreactivity was expressed as a percentage of the total PSD-95 puncta for each rat in the study (values were then normalized to the within-cohort ischemic control rat mean). The C group had a higher percentage of PSD-95 puncta colocalized with pCofilin than did either N or S groups; differences in pCof+ puncta colocalized with pCofilin between TTI versus non-TTI or C groups were also significant. Data were expressed as mean ± SEM. #* p*<0.05 vs. non-TTI and C groups; *∗∗∗p*<0.001 vs. N and S groups.

In the contralateral prelimbic cortex, the number of synapses positively expressed by pCofilin (pCof+) was significantly higher in group C compared to groups N and S (*∗∗∗* p < 0.001); there was no difference between group C and subgroup non-TTI. When compared to subgroup non-TTI and group C, respectively, the number of pCof+ synapses in subgroup TTI was higher (# p < 0.05); see Figure 3(a). The number of synapses positively expressed by PSD-95 was not distinct in each group; see Figure 3(b). The number of synapses positively expressed by pCof+PSD-95 was significantly increased in group C compared with groups N and S (*∗∗∗* p < 0.001). There was no significant difference between group C and subgroup non-TTI. The number of pCof+PSD-95 was greatly higher in subgroup TTI compared to subgroup non-TTI and group C, respectively (## p < 0.01); see Figure 3(c).

### 3.4. PCofilin-Positive Spines Were Correlated with the Enlargement of Synapses

**Figure 4:**
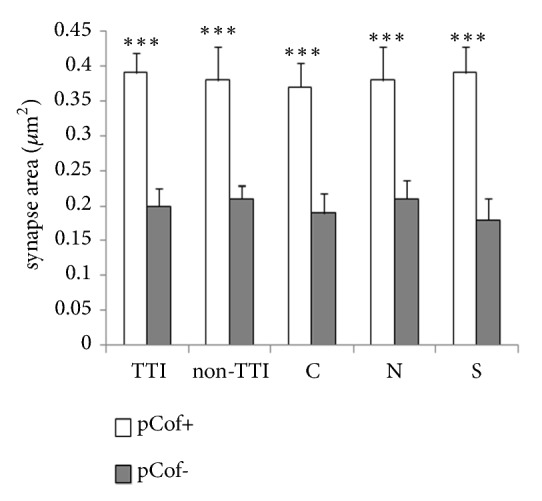
PSDs on pCof+ spines were larger than those on pCof- neighbors. Data were expressed as mean ± SEM. *∗∗∗p < *0.001.

PSD-95 is a protein that is specific to and uniformly distributed within PSDs of excitatory synapses. This experiment showed that the total number of PSDs was not affected by moxibustion intervention and MCAO model. However, 3-D measurement of PSD-95 showed that PSD-95 positive profiles were larger on pCofilin positive (pCof+) spines than on pCofilin negative (pCof-) spines (*∗∗∗* < 0.001). It follows from this result that MCAO model by increasing the number of pCofilin-enriched spines also increased the number of spines with large synapses, and suspended moxibustion exercise to MCAO rats with tail temperature increase had enhanced this effect; see Figure 4.

## 4. Discussion

Through our clinic practice, we found that certain subhealthy individuals or patients were able to sense a certain special heat in the body while exposed to suspended moxibustion exercise, which was not part of moxibustion heat radiation. The sense of the special heat was described as heat expansion, heat penetration, and heat transmission, and those were not clarified by neuroanatomy or physiology. In clinic practice, suspended moxibustion exercise with special heat awareness has demonstrated good healing over time, and the sense of moxibustion-induced special heat has drawn more attention in clinic practice. Accordingly, we named the sense as heat-sensitization responses [1]. Tracing back to classic traditional Chinese medicine texts, the sense of moxibustion-induced special heat was mentioned [11] such that moxibustion-induced heat was sensed by the chest through rectal path when moxibustion exercise to the anus. Based on our studies and clinic practice, moxibustion-induced heat-sensitization responses are characterized by three traits [1]: (1) it rarely occurred under normal physiological conditions (<10%), but the incidence rate was higher under pathological conditions (>70%); (2) it phased out along the attenuation of medical conditions; (3) moxibustion demonstrated a great curative effect when it was sensed by the recipient. These three traits feature the sense variation; it relates to the state of a disease and also indicates treatment efficacy of moxibustion.

The current theory is not sufficient to explain the mechanism of heat-sensitization responses. For example, a patient with fibromyalgia in the back of the head and neck was exposed to moxibustion exercise on the acupoint Zhì yáng (GV9) (Supplementary Figure S3). The sense of heat was transmitted along the spine or paraspinal muscles to the muscles of posterior part of the head and neck. That is to say, the heat sensation of the lower part of the spinous process of the seventh thoracic spine can be transmitted to the cervical vertebra. However, the classical sensory conduction pathway has clearly pointed out that the process of the brain feeling the stimulation of a certain part of the body is that the stimulating signal received by sensory device is ascended through the spinal cord or trigeminal or medial colliculus system to the thalamus, by which the nature of the stimulation is judged and then projected to the central posterior gyrus primary sensory cortex [12]. Regarding this sense of moxibustion-induced heat transmission, noticeably, the remote parts of these moxibustion sites were not exposed to the heat stimulation. However, the recipient was able to sense the trans-segmental heat in the distant parts. Xie et al. [4] used fMRI to study the responses and found that the brain function regulation of heat-sensitization responses did not coordinate through a single brain region but a network composed of multiple brain regions, especially in the prefrontal and island cortices. Liao et al. [13] used high-density electroencephalogram (EEG) to test the EEG characteristics of moxibustion-induced heat-sensitization responses. They found that the power spectral density of *α* and *β* waves increased greatly. The average power topology showed that *ϭ* and *β* waves changed most significantly in the central frontal region, while the change of *α* band was across the whole global. In addition, heat-sensitization responses and non-heat-sensitization responses in the process of moxibustion showed different patterns of EEG activity. The former can induce phase coherence increased in the range of *ϭ* and *β*. Both studies show that the brain is involved in heat-sensitization responses. According to the brain plasticity, its loop connection and function vary under certain medical conditions. It is worthy to further explore the relations between neuroplasticity and heat-sensitization responses of moxibustion.

Synaptic plasticity refers to changes in synaptic transmission efficiency and synaptic structure under certain conditions. Long-term potentiation (LTP) is a key manifestation of synaptic plasticity [14]. The change of synaptic structure requires the participation of structural protein actin fiber, and the change of action fiber is regulated by Cofilin protein. The phosphorylation of Cofilin directly affects the degree of aggregation of action fiber, which in turn affects synaptic structure and dendritic spines size [15]. That being the case, phosphorylation of Cofilin is considered a marker of LTP* in vivo* [6], and detection of phosphorylation of Cofilin can reflect synaptic plasticity. Although LTP can be assessed by conventional electrophysiological method, this method would produce additional experimental traumas in MCAO rats which might affect heat-sensitization response. Hence, we need to reduce additional experimental traumas in MCAO rats as much as possible. Besides, the stimulated acupoint DU14 is close to the brain. Recording LTP by conventional electrophysiological method might be affected by the heat from moxibustion. In this study, we observed the phosphorylation of Cofilin in the synapse of the brain to determine whether its neuroplasticity relates to the heat-sensitization responses of moxibustion.

PrL of mPFC was selected as the observation region of brain tissue by reasons such as the fact that PrL is the key region of mPFC, a widely studied brain region that has extensive and complex connections with other brain regions, and plays an important role in cognitive processes, decision-making tasks, emotional regulation, pain regulation, and many other undefined functions [16, 17]. Besides, fMRI and high-density EEG studies suggest that heat-sensitization responses were associated with activity changes in the frontal lobe [4, 13]. In addition, in order to minimize the influence of MCAO model on the mPFC, this study chose the contralateral mPFC as the observation area. After the MCAO model was made, the MCA blood supply area was necrotic. The surroundings of infarct neural tissue were affected by physiological and biochemical changes inflammatory mediators and apoptosis, especially in the lateral prefrontal cortex. When the infarct size was large, it would directly affect the mPFC.

In our previous experiments, we observed that the temperature in certain rats' tail midpoints rose more than 2°C when moxibustion exercise was exerted upon the acupoint DU14 of cerebral ischemia-reperfusion injury rats [7, 10]. We also found that the cerebral infarction volume and neurological deficit score were greatly improved in these rats with tail temperature significant increase. It also has better antiapoptosis and anti-inflammatory effects [10, 18]. Previous studies have demonstrated that this effect of tail temperature increase was not caused by thermal radiation [7]. Accordingly, the results give credit for the fact that the increase of tail temperature caused by moxibustion exercise upon DU14 acupoint in stroke rats should be similar to the clinical manifestation of heat-sensitization responses [7]. The rats' MCAO model with tail temperature increase induced by moxibustion upon DU14 in this study can be applied to study into the mechanism of heat-sensitization responses, instead of language communication in clinic practice.

The results of this study show that the MCAO model itself (representing the disease state) significantly increased phosphorylation of Cofilin protein in the PrL of rats. It indicates that the disease state could induce LTP-like plasticity changes in the PrL of rats. Many studies have confirmed that disease states promote changes in certain cortical functions. For example, Rainville et al. found that visceral pain changed activities of the anterior cingulate cortex [19]. The links between human body and certain regions of the brain under pathological conditions are a ubiquitous phenomenon, and more studies are needed. Should the disease state itself cause functional or structural changes in certain areas of the brain, a new neural circuit may generate. This change may be due to biological evolution, so that the body can be regulated endogenously under pathological conditions. Our study also showed that the number of pCofilin positive expressions in subgroup TTI was significantly higher than that in subgroup non-TTI or group C. This indicates that heat-sensitization responses enhanced the effect of LTP-like plasticity in PrL under pathological condition. Since heat-sensitization responses were observed closely relating to curative effect of moxibustion exercise, it could be inferred that LTP in PrL is a key role in the recovery of neurological function for stroke rats. Increase of LTP of PrL over moxibustion exercise would provide a promising opportunity for cerebral ischemic stroke treatment. In addition, an important synaptic protein PSD-95 was also observed in this study. Its size was proved as an important indicator of the size of synaptic dendritic spines [20]. Synaptic structural remodeling is accompanied with the size change of PSD-95 [5, 20]. The results showed that there was no significant change in the total number of PSDs among each group, but the PSDs dendritic spines with pCofilin positive expression were larger than those with pCofilin negative expression. It follows from the result that MCAO model by increasing the number of pCofilin-enriched spines also increased the number of spines with large synapses, and heat-sensitization responses in rat's model characterized by rat's tail temperature increase had enhanced this effect.

To sum, rats' MCAO model induced phosphorylation of Cofilin in PrL of mPFC and enlarged synapses. Moxibustion-induced heat-sensitization responses complemented this aggregation. The increase of pCofilin positive spines in PrL represents LTP of PrL. Therefore, we point out that LTP of PrL was attributed to heat-sensitization responses. It is worthy to run a further study on exploring a way simulating the heat sensitive moxibustion to enhance LTP of PrL for cerebral ischemic stroke.

## Data Availability

The data used to support the findings of this study are available from the corresponding author upon request.

## Abbreviations

mPFC: Medial prefrontal cortex
PrL: Prelimbic cortex
TTI: Tail temperature increase
LTP: Long-term potentiation
pCofilin: Phosphorylated Cofilin
EEG: Electroencephalogram
fMRI: Functional magnetic resonance imaging
MCAO: Middle Cerebral Artery Occlusion.

## Supplementary Materials

Supplementary Materials Supplemental Figure S1: clinical operation of suspended moxibustion. Supplemental Figure S2: the rat's tail temperature increase when acupoint DU14 was exposed to suspended moxibustion. Supplemental Figure S3: the sense of heat in a patient with fibromyalgia was transmitted along the spine or paraspinal muscles to the muscles of posterior part of the head and neck when acupoint GV9 was exposed to suspended moxibustion.
