# Supplementary figures and images for: Long-Term Potentiation of Prelimbic Cortex Ascribed to Heat-Sensitization Responses of Moxibustion
Source: Evid Based Complement Alternat Med. 2019 Jul 25;2019:9465181. doi: 10.1155/2019/9465181 (PMC6683778; doi:10.1155/2019/9465181)

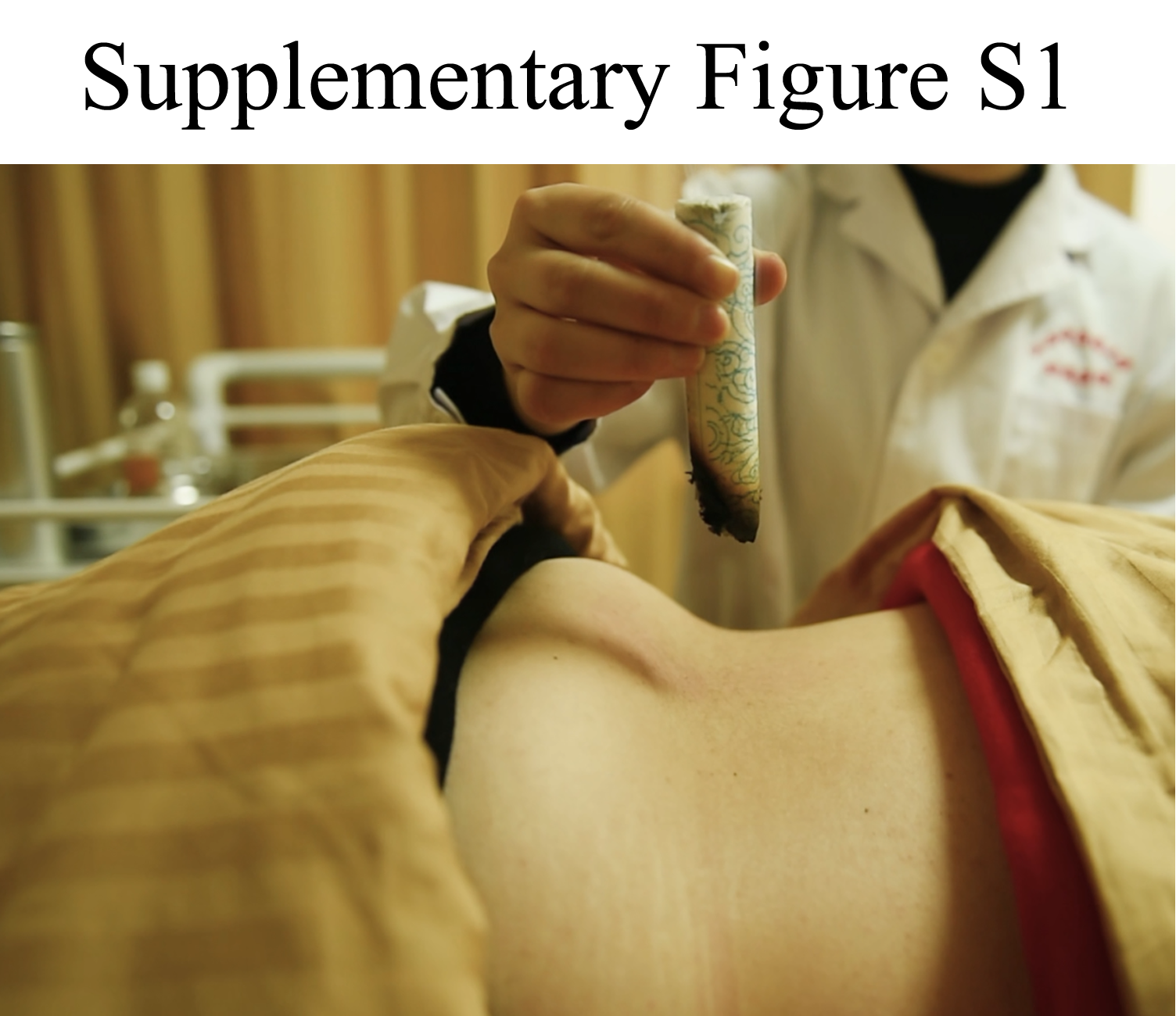


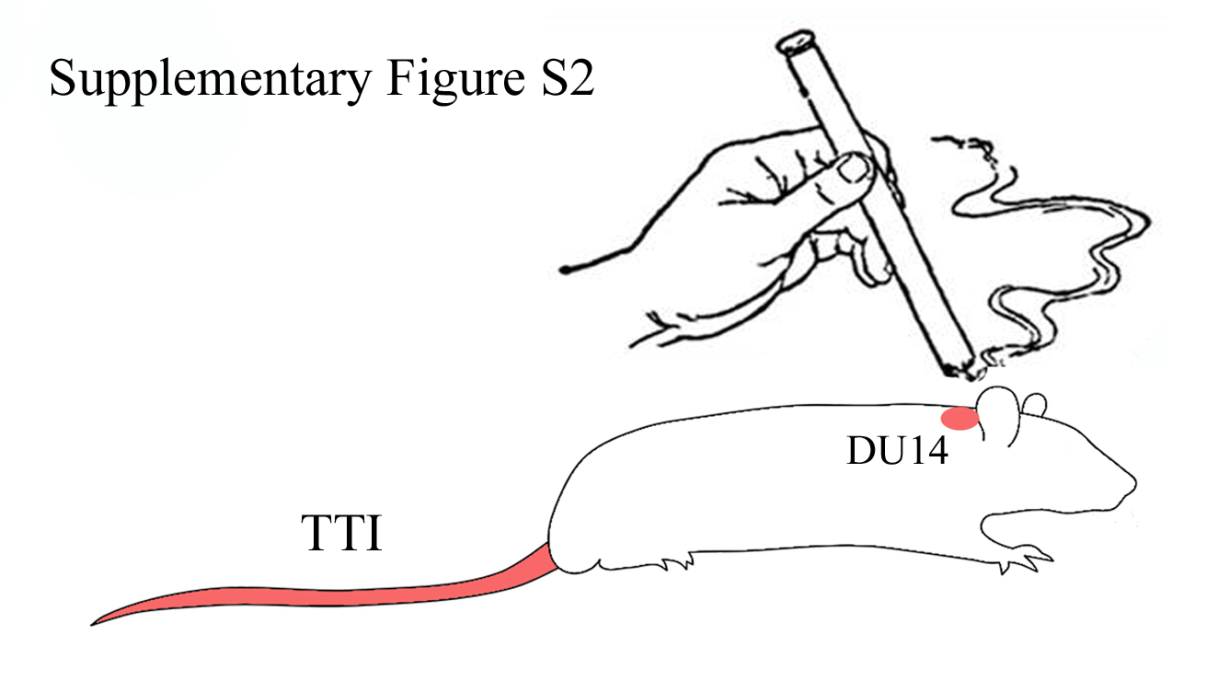


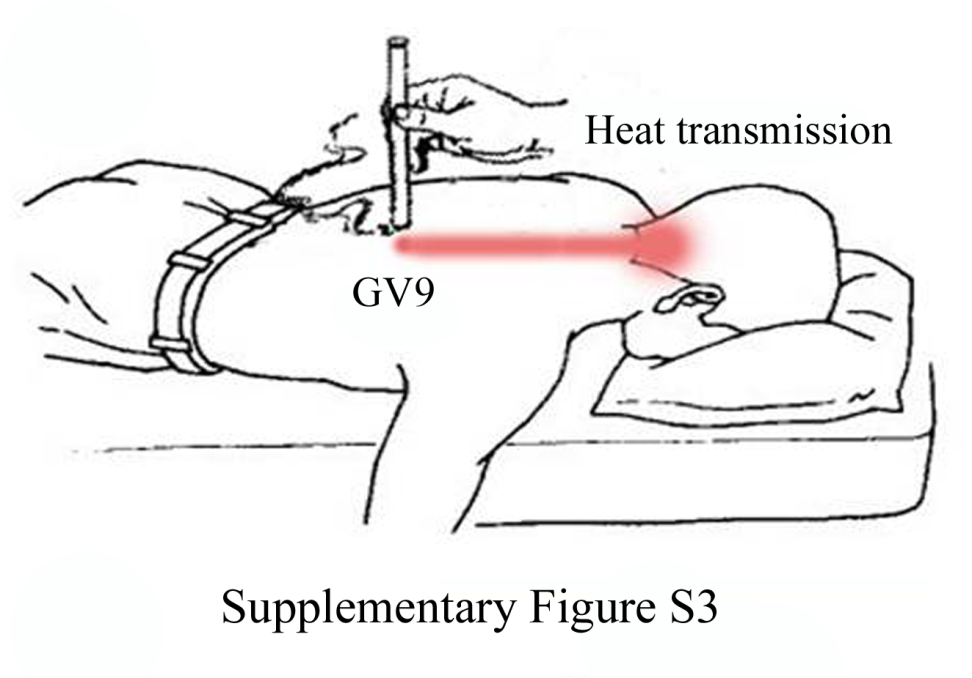

Supplement: Supplementary Materials — Supplemental Figure S1: clinical operation of suspended moxibustion. Supplemental Figure S2: the rat's tail temperature increase when acupoint DU14 was exposed to suspended moxibustion. Supplemental Figure S3: the sense of heat in a patient with fibromyalgia was transmitted along the spine or paraspinal muscles to the muscles of posterior part of the head and neck when acupoint GV9 was exposed to suspended moxibustion. [file 9465181.f1.docx]
